# The role of retinoic acid signaling in starfish metamorphosis

**DOI:** 10.1186/s13227-018-0098-x

**Published:** 2018-04-21

**Authors:** Shumpei Yamakawa, Yoshiaki Morino, Masanao Honda, Hiroshi Wada

**Affiliations:** Graduate School of Life and Environmental Sciences, University of Tsukuba, Tsukuba, Ibaraki 305-8572 Japan

**Keywords:** Retinoic acid signaling, Metamorphosis, Echinoderm, Starfish

## Abstract

**Background:**

Although retinoic acid (RA) signaling plays a crucial role in the body patterning of chordates, its function in non-chordate invertebrates, other than its mediation of environmental cues triggering metamorphosis in cnidarians, is largely unknown. We investigated the role of RA signaling in the metamorphosis of starfish (Echinodermata).

**Results:**

We found that exogenous RA treatment induced metamorphosis in starfish larvae. In contrast, inhibitors of RA synthesis and RA receptors suppressed metamorphosis triggered by attachment to a substrate. Gene expressions of the RA signaling component were detected in competent larvae.

**Conclusions:**

This study provides insight into the ancestral function of RA signaling, which is conserved in the metamorphosis of cnidarians and starfish.

**Electronic supplementary material:**

The online version of this article (10.1186/s13227-018-0098-x) contains supplementary material, which is available to authorized users.

## Background

Retinoic acid (RA) plays a critical role in the body patterning of chordates, such as the anterior–posterior patterning of the central nervous system and pharyngeal arches [1–3]. RA synthesized by retinal dehydrogenase (Raldh) regulates downstream gene expression through binding to the retinoic acid receptor (RAR) and the retinoid x receptor (RXR) heterodimer nuclear receptor. Although RXR was identified in various metazoan taxa including cnidarian, arthropod, and nematode [4], Raldh and RAR, as well as cytochrome P450 26 (CYP26), which degrades RA, had been described only in chordates, and thus RA signaling was thought to be specific to chordates, and the acquisition of the gene families for RA signaling was thought to be a key step in the evolution of the chordate body plan [3]. However, Cañestro et al. reported that gene families of Raldh, RAR, and CYP26 are encoded in the genomes of non-chordate deuterostomes [5]. Following additional genomic surveys of other invertebrates, the origin of RA signaling was pushed back to the common ancestor of metazoans [6, 7].

Despite these genomic surveys, the function of RA signaling in non-chordate deuterostomes remains largely unknown, other than the observation of pseudopodial cable growth on micromere-delivered cells from *Hemicentrotus pulcherrimus* after RA treatment [8]. Sciarrino et al. reported that RA treatment on *Paracentrotus lividus* did not induce specific phenotype except for delaying development [9]. Although Marlétaz et al. suggested that the promoter region of sea urchin *Hox* genes had an RA response element [3], no additional evidence of the effect of RA on *Hox* gene expression has been reported.

Insight into the ancestral function of RA signaling can be gained from the study of cnidarians, a basal lineage of animals. Fuchs et al. showed that RA signaling is involved in the life cycle transition of cnidarians [10]. Medusozoan cnidarians have two distinct life cycle phases. Fertilized eggs first develop into planula larvae and then, through further development, become sessile polyps. When the polyps receive environmental stimuli, such as a temperature change, they metamorphose into medusae [10]. Fuchs et al. reported that RA is an internal regulator of this process and suggested that RA works through binding with RXR [10]. Because RXR is known to be involved in the metamorphosis of insects and frogs [11–16], RA signaling was suggested to also have a role in the life cycle regulation of metazoans.

Molecular mechanisms for metamorphosis have been investigated in echinoderms, primarily echinoids [17–28]. In the sea urchin *Strongylocentrotus purpuratus*, larvae acquired competence to metamorphose 4.5–6 weeks after fertilization [25] and commenced metamorphosis in response to substrates such as algal sheets [29]. Previous studies suggested that thyroid hormone (TH) and histamine (HA) functioned as modulators of larval growth and the competent state [19–21, 24–27]. Although several works also revealed nitric oxide (NO) signaling functioned as a negative regulator upon settlement [17, 18], any role of RA signaling in metamorphosis is not reported in any taxa of echinoderm.

Here, we investigate the role of RA signaling in asteroid metamorphosis. We used the starfish *Patiria pectinifera*, as it acquires competence to metamorphose earlier than sea urchin species (about 1 week after fertilization). We show that exogenous RA treatment induced larval body absorption and juvenile rudiment development in competent larvae. Conversely, the inhibition of endogenous RA synthesis or binding of RA to RAR blocked metamorphosis. In addition, genes related to RA signaling were expressed in the juvenile and larval stages. These results suggest that RA signaling acts as a regulator of metamorphosis after settlement.

## Methods

### Sampling and culture

We collected adult specimens of *P. pectinifera* around the mainland of Japan: Manaduru (Kanagawa Prefecture), Asamushi (Aomori Prefecture), and Hiraiso (Ibaraki Prefecture). Fertilization and embryo rearing were performed from described previously [30]. From 2 days post-fertilization (dpf), the larvae were fed *Chaetoceros calcitrans* (purchased as Sun-culture: Marine Tec, Aichi) by rearing in seawater with 50,000 cells/ml. We changed seawater with fresh algae every other day.

### Reagent treatment

The RA signaling pathway was activated by exogenous all-trans RA (Sigma-Aldrich, St Louis, CAS number: 302-79-4). RA signaling was inhibited by the Raldh inhibitor *N*,*N*-diethylaminobenzaldehyde (DEAB, Tokyo Chemical Industry, Tokyo, CAS number: 120-21-8) or the RAR antagonist RO41-5253 (RO, Focus Biomolecules, Pennsylvania, CAS number: 144092-31-9). We prepared 100 mM, 1 M, and 50 mM stocks of RA, DEAB and RO, respectively, in dimethyl sulfoxide (DMSO). Larvae were incubated in 2 ml artificial seawater containing 2 µl of reagents or DMSO in 12-well plates at 22 °C. For the treatment without a substrate, 10 larvae were incubated in one well. In the experiments conducted to induce metamorphosis with a substrate, a single larva was cultured in one well to identify individuals. We observed metamorphosis proceed in the same manner in these densities of larvae. Coral sand from tanks of adult *P. pectinifera* was used as a substrate for *P. pectinifera* experiments [31]. For cases in which drug treatment continued for more than 2 days, we changed the seawater with the same concentration of drugs every other day.

**Fig. 1:**
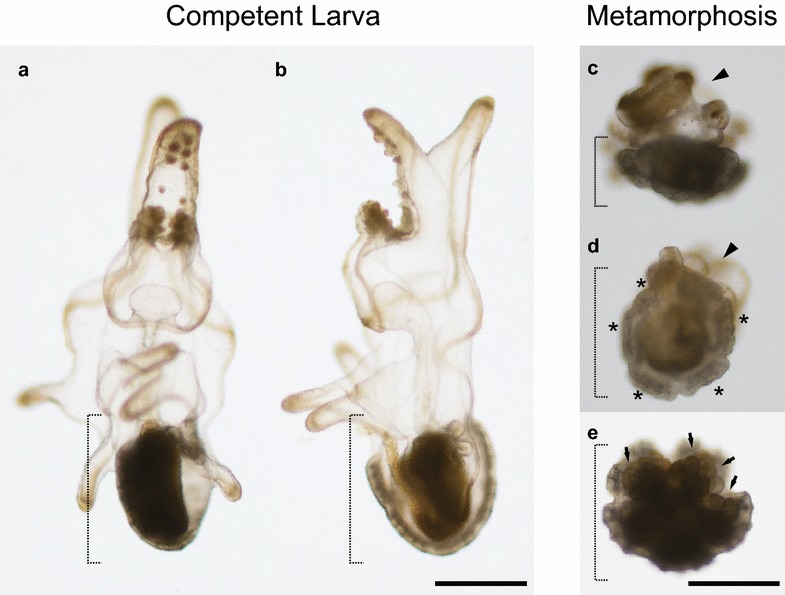
Competent and metamorphosis larva of *P. pectinifera.* **a**, **b** Ventral (**a**) and left side (**b**) view of competent 20-dpf larva. **c**–**e** Metamorphosis larva in 24 h after settlement. **c** Larval ventral side of metamorphosis larva. **d**, **e** attend on aboral and oral side of juvenile rudiment, respectively. Dot lines indicate juvenile rudiment and asterisk marks do developed juvenile arms. Arrow heads point absorbed larval body and arrows do primary podia. Size of scale bar is  250 µm

We considered the ability to induce metamorphosis to have been achieved when larval body absorption and the development of juvenile rudiment with primary podia were observed (Fig. 1). We considered larvae adhered to the substrate with brachiolar arms to be settled. In order to test effects of those reagent, we set several conditions in the experiments and assumed these conditions as main factors: density of DEAB (0, 100 and 300 µM) and presence/absence of RO (1 µM) for settlement and metamorphosis, and combination of density of RA (0.1 and 1 µM) and presence/absence of RO (3 µM) for metamorphosis. Each data was obtained from three batches of different parent pair. Within batches, treatments of density of DEAB and presence/absence of RO were repeated thrice and twice, respectively. In total, we used 324 and 324 individuals for experiments of density of DEAB in settlement and metamorphosis, respectively, 144 and 144 for those of presence/absence of RO in settlement and metamorphosis, respectively, and 240 for those of combination of density of RA and presence/absence of RO in metamorphosis.

### Statistical analysis

The metamorphosis ratio was calculated by dividing the number of metamorphosis commenced larvae by the number of treated larvae. In the case of reagent treatment with substrate, metamorphosis ratio was calculated by dividing the number of metamorphosed larvae by the number of settled larvae.

We used ANOVA to evaluate differences of reagent treatments on the metamorphosis or settlement, assuming above conditions and batches as main factors and “blocks,” respectively, because preliminary tests in some treatments revealed slight, but not essential, differences among batches. If we had two factors, we assessed an interaction of the two factors. Also, multiple comparison was performed by Tukey’s honestly significant difference test. The R statistical software was used for all statistical analyses [32].

As to the assumption of normal distribution, we use Kolmogorov–Smirnov test and confirmed no significant difference from the assumption of normality. Also, Bartlett’s test was used to test homogeneity of variances prior to ANOVA. In a few experiments, we found significant violations of the equal variance assumption and conducted Welch’s one-way ANOVA to test main effects without the assumption of equality. However, there were no differences in statistical conclusions between the main factor of ANOVA and the Welch’s method. We thus used ANOVA with assumption of equal variance in the following analyses because we needed multi-dimensional analyses simultaneously and because ANOVA is robust to deviations from equal variance, especially if numbers of sample are equal [33], and normality assumptions [34].

### Identification of genes related to RA signaling in starfish

To gather sequence of RA signaling related genes, we performed transcriptome analysis on samples of blastula (13 h post-fertilization), brachiolaria larva (14 dpf) and adult epidermis in starfish *P. pectinifera*. Each sample was collected from three biological replicates: The samples of blastula and brachiolaria larva were collected from progeny of three distinct pairs of parents, and the adult epidermis was collected from three mother specimens. Total RNA was extracted using TRIzol reagent (Life Technologies, Carlsbad, CA, USA) and treated with DNase and cleaned up using the RNeasy kit (Qiagen, Hilden, Germany). Paired-end libraries (200 bp) were prepared using a TruSeq RNA Sample Preparation Kit (Illumina). Sequencing on the Hiseq 2000 platform (Illumina, San Diego, CA, USA) was performed at the National Institute of Basic Biology (NIBB) The raw reads were deposited in the DDBJ Sequence Reads Archives (DRA006662). The low-quality reads were filtered using the NGS QC Toolkit57 [35]. De novo assembly was conducted by Trinity58 (version: trinityrnaseq_r20140717) [36].

We recovered nucleotide sequences coding for *raldh*, *rar* and *rxr* from gene models assembled from our de novo transcriptome data of larval stages for *P. pectinifera*. The orthologies of these genes were confirmed by molecular phylogenetic analysis (Additional file 1: Fig. S1, Additional file 2: Fig. S2). All sequences are shown in Additional file 3: Table S1 and aligned using MAFFT ver. 7 (https://mafft.cbrc.jp/alignment/server/) with the default parameters [37]. Amino acid sites for tree construction were selected by trimAl using a gap threshold value of 0.8 [38]. A best-fitting amino acid substitution model and a maximum likelihood tree were inferred using RAxML 8.2.0 [39]. Confidence values were calculated after 1000 bootstrap runs.

### Whole-mount in situ hybridization

cDNA was obtained by reverse transcription from larval RNA using PrimeScript 1st strand cDNA Synthesis kit (Takara, Shiga, Japan). Additional file 4: Table S2 shows the sequences of the primers used for the amplification of *raldha*, *raldhb*, *raldhc*, *rar*, and *rxr*. We used 40-bp reverse primers including a 20-bp T3 promoter sequence to synthesize Dig-labelled RNA probes for in situ hybridization. Dig-labelled RNA probes for *raldha*, *raldhb*, *raldhc*, *rar*, and *rxr* were transcribed from PCR products using T3 RNA polymerase (Roche, Mannheim, Germany). In situ hybridization was performed as described previously [40].

## Results

### Competence for metamorphosis was acquired around 8 dpf in *P. pectinifera*

As in other echinoderm species, the transition from larval form to adult form proceeds via multiple steps in starfish. Development of the adult rudiment commences well before larvae acquire competence. Adult skeletogenesis was observed in penta-radial manner as early as 6 dpf, when development of brachiolar arms commences. Development of the brachiolar arms is tightly linked with the acquisition of competence. Murabe et al. indicated that adhesive papillae on the brachiolar arms sense environmental cues such as proper substrate for promoting metamorphosis [31]. When competent larvae attach to proper substrate, they initiate metamorphosis. The metamorphosis is observed as absorption of larval body and development of pentaradial juvenile body with primary podia (the precursor structures of tube feet), which occur within 24 h after settlement (Fig. 1).

Although development of the brachiolar arms commences at 6 dpf, we do not know when the competence to receive cues for this metamorphosis is acquired. Thus, we first sought to identify the stage at which *P. pectinifera* larvae become competent to metamorphose upon attachment to a substrate.

**Fig. 2:**
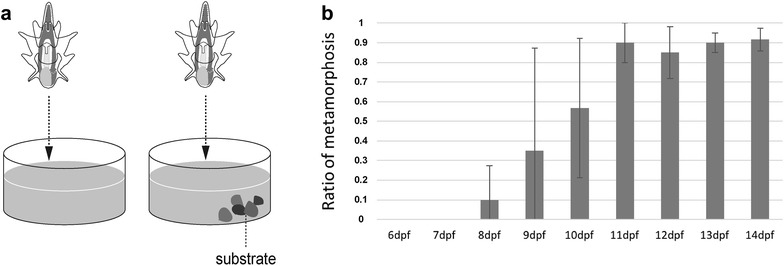
Duration until *P. pectinifera* became competent. **a** Schematic experiment sets without or with substrate (left and right, respectively). Without external substrate, larva did not settle and complete metamorphosis. **b** Ratio of metamorphosis completion 24 h after adding substrate (*n *= 20 from each three batch)

Here we examine the competence by testing whether larvae initiate metamorphosis upon attachment to coral sand taken from a tank of adult *P. pectinifera*. Note that we are not certain the coral sand was the most favorite substrate, and thus, larvae may initiate metamorphosis earlier when they attach to other substrates. We introduced coral sand to the wells of larvae of various ages (Fig. 2a) and counted the number of larvae that completed metamorphosis within 24 h. Although larvae younger than 7 dpf showed no response to the introduced substrate, some 8-dpf larvae (0, 6, 0 of 20 larvae from each batch) completed metamorphosis (Fig. 2b). The remaining larvae did not settle or metamorphose, but crawled around the substrate and attached repeatedly. This behavior was identified as characteristic prior to settlement and metamorphosis [31]. More than 85% of larvae older than 11 dpf that were provided with substrate completed metamorphosis (Fig. 2b). These results indicate that larvae became competent to metamorphose upon attachment to coral sand at approximately 8 dpf. Of note, because larvae start feeding at 2 dpf, their growth and developmental rates depend on the nutrition level and are thus not uniform. Therefore, the reported age of the larvae is a rough estimate of the developmental stage.

### Exogenous RA treatments with competent *P. pectinifera* larvae induced metamorphosis

**Fig. 3:**
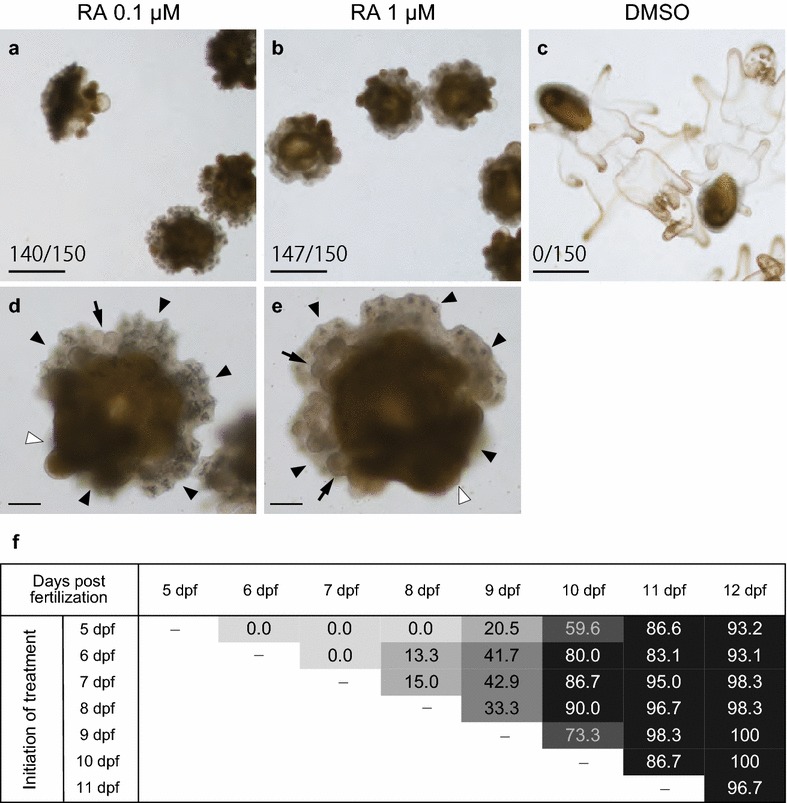
Effects on metamorphosis by all-trans RA or DMSO treatments in *P. pectinifera.* **a**–**e** Metamorphosis induction of 14-dpf larvae by exogenous all-trans RA (0.1 or 1 µM) treatment (*n *= 50 from each three batch). **a**, **b**, **d**, **e** Induced juvenile of 96 h after RA 0.1, 1 µM treatment, respectively. **c** DMSO-treated larvae (96 h after treatment). Arrow heads indicate developed juvenile rudiment, white arrowheads point out absorbed larval body, and arrows show primary podia. The scale bars in **a**–**c**, **d**–**e** display 250, 50 µm, respectively. The number in figure show the ratio of metamorphosed larvae/treated larvae. **f** Temporal effects on metamorphosis induction of all-trans RA (1 µM). Numbers in figure show percentage of metamorphosed specimen (*n *= 20 from each three batch in each treatment) and background colors are depended on their percentage and divided into five darkness levels, meaning 0, 0–25, 25–50, 50–75 and 75–100%. Vertical axis indicates days after treatment and horizontal axis indicates days after fertilization

We then investigated the function of RA signaling in the metamorphosis of *P. pectinifera*. Most competent larvae treated with 1 µM all-trans RA at 14 dpf showed larval body absorption and juvenile rudiment development with primary podia (50, 50, and 47 of 50 larvae from each batch; Fig. 3b, e). Larval body absorption was observed immediately after RA treatment, and metamorphosis into a pentaradial juvenile body with primary podia was observed within 24 h (Fig. 3b, e). A similar effect was observed when larvae were treated with a lesser amount (0.1 µM) of RA (50, 50, and 40 of 50 larvae from each batch; Fig. 3a, d), whereas no larva showed any sign of larval body absorption or juvenile body development with the DMSO control (0, 0, and 0 of 50 larvae from each batch; Fig. 3c).

These results indicate that all-trans RA has the potential to induce larval body absorption and juvenile rudiment development when added to competent larvae at 14 dpf.

### The effect of RA treatment on metamorphosis was limited after larvae became competent

We investigated whether RA can also affect the timing of larval competence to respond to the metamorphosis cue. No larva treated with 1 µM all-trans RA at 5–7 dpf commenced metamorphosis, as judged by larval body absorption and juvenile rudiment development (Fig. 3f). RA treatment of larvae younger than 8 dpf had no apparent effect on morphology or behavior before 8 dpf. However, some of these larvae cultured in seawater with RA commenced metamorphosis at 8 dpf (Fig. 3f). Regardless of when the RA treatment started, larvae responded to RA and metamorphosed at 8 or 9 dpf, which is comparable with the stage at which larvae acquire competence to metamorphose during normal development (Figs. 2, 3f). Most larvae that were treated with 1 µM RA after 8 dpf commenced metamorphosis within 24 h (Fig. 3f). These timelines are similar to those induced by a substrate. Above results indicate that RA does not affect the development of competence for metamorphosis, but rather functions as a cue to commence metamorphosis when added to competent larvae.

### RAR activation was required for the initiation of metamorphosis

**Fig. 4:**
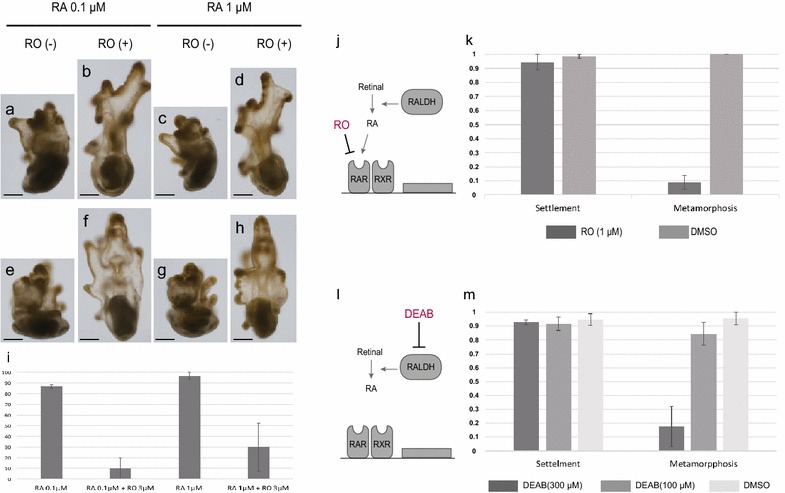
Effects on settlement or metamorphosis by RO or DEAB treatments in *P. pectinifera.* **a**–**i** RO 3 µM treatment inhibited metamorphosis of 14-dpf larvae induction by RA 1 µM treatments (*n *= 20 from each three batch). **a**–**d** Left side of specimen at 24 h after treatment and **e**–**h** does ventral or dorsal side of these. Each specimen is taken from following treatment, **a**, **e** RA 0.1 µM, **b**, **f** RA 0.1 µM with RO 3 µM, **c**, **g** RA 1 µM and **d**, **h** RA 1 µM with RO 3 µM. The scale bars in **a**–**h** display 125 µm. **i** indicates metamorphosis ratio of 24 h after treatments. **j**, **k** Effects of RO in settlement and metamorphosis (*n *= 24 from each three batch). **j** Schematic machinery of inhibition of RA signaling by RO. **k** Settlement and metamorphosis ratio of 14-dpf larvae in 24 h after RO 1 µM treatment with substrate (*n *= 72 from three batches). **l**, **m** Effects of DEAB in settlement and metamorphosis (*n *= 36 from each three batch). **l** Schematic machinery of inhibition of RA signaling by DEAB. **m** Settlement and metamorphosis ratio of 14-dpf larvae in 24 h after DEAB 100, 300 µM treatment with substrate

To examine whether RA functions as an internal mediator of signaling to a metamorphosis cue, we treated larvae with RO, known to be the selective RARα antagonist (Fig. 4j). As shown previously, most 14-dpf larvae commenced metamorphosis when treated with 0.1 µM or 1 µM RA (Fig. 4a, c, e, g, i). We found that the presence of RO significantly suppressed metamorphosis rate (*P* < 0.001, ANOVA, see Fig. 4, Additional file 5: Table S3) although effect of RA and interaction of RO and RA were not significant (*P* = 0.182 and 0.633, respectively, ANOVA). ANOVA revealed a significant effect of RO on the ratio of metamorphosed larvae (*P* = 0.182, 0.000, 0.632 and 0.108 in RA, RO, RA*RO, and batch, respectively, ANOVA).

These results suggest that exogenous RA drives metamorphosis through binding to RAR. Therefore, we investigated whether RAR plays any role when larvae respond to a substrate and commence metamorphosis. We treated competent larvae with 1 µM RO at 14 dpf. As shown above, more than 90% of 14-dpf larvae responded to the introduction of coral sand and commenced metamorphosis (Fig. 4k). However, when 1 µM RO was added together with the substrate to 14-dpf larvae, metamorphosis was significantly inhibited (*P* = 0.000 and 0.632 in RO and batch, respectively, ANOVA; Fig. 4k right, Additional file 6: Table S4). Interestingly, in contrast to metamorphosis, RO treatment did not affect the number of larvae attached to the substrate (*P* = 0.256 and 0.058 in RO and batch, respectively, ANOVA; Fig. 4k left, Additional file 7: Table S5). These results are consistent with the idea that RAR functions as an internal mediator of the signaling cue for metamorphosis received by the adhesive papillae [31].

### Endogenous RA synthesis was essential for metamorphosis after settlement

To assess whether endogenous RA synthesis was required for metamorphosis, we treated 14-dpf larvae with DEAB, a known inhibitor of Raldh (Fig. 4l). We cultured larvae in seawater with 100 or 300 µM DEAB and coral sand as a substrate. DEAB-treated larvae crawled around the substrate surface, and most settled to the substrate with the brachiolar arms, as normal competent larvae do. The ratio of settled larvae in the DEAB treatments did not differ significantly (*P* = 0.728, ANOVA; Fig. 4m left, Additional file 8: Table S6), although significant difference was observed among batches (*P* = 0.010 in batch, ANOVA). However, DEAB significantly inhibited metamorphosis after settlement. Significance of DEAB was found in metamorphosis (*P* = 0.000 and 0.001 in DEAB and batch, respectively, ANOVA; Fig. 4m right, Additional file 9: Table S7). The metamorphosis rate was slightly lower in the 100 µM DEAB treatment than in the DMSO treatment. Inhibition of 300 µM DEAB was significantly effective with DMSO and 100 µM DEAB treatments (*P* = 0.000 and *P* = 0.000, Tukey’s test, respectively), although there was no significant difference between the latter two in metamorphosis (*P* = 0.624, Tukey’s test). These results indicate that endogenous RA synthesis was essential for the commencement of metamorphosis after receipt of the signaling cue by the adhesive papillae, whereas it was not essential for settlement on the substrate.

### Temporal and spatial expression patterns of genes related to RA signaling in larvae

The results reported above suggest that RA signaling functions as an endogenous mediator of a metamorphosis cue upon settlement. To support this idea, we surveyed RA signaling genes in our transcriptome data for *P. pectinifera*. We investigated their orthologues by construction of phylogenetic trees (Additional file 1: Fig. S1, Additional file 2: Fig. S2). We identified three *raldh* genes in our transcriptome data: *raldha, raldhb and raldhc* (Additional file 1: Fig. S1). Single genes for *rar* and *rxr* were also identified from our transcriptome data (Additional file 2: Fig. S2).

**Fig. 5:**
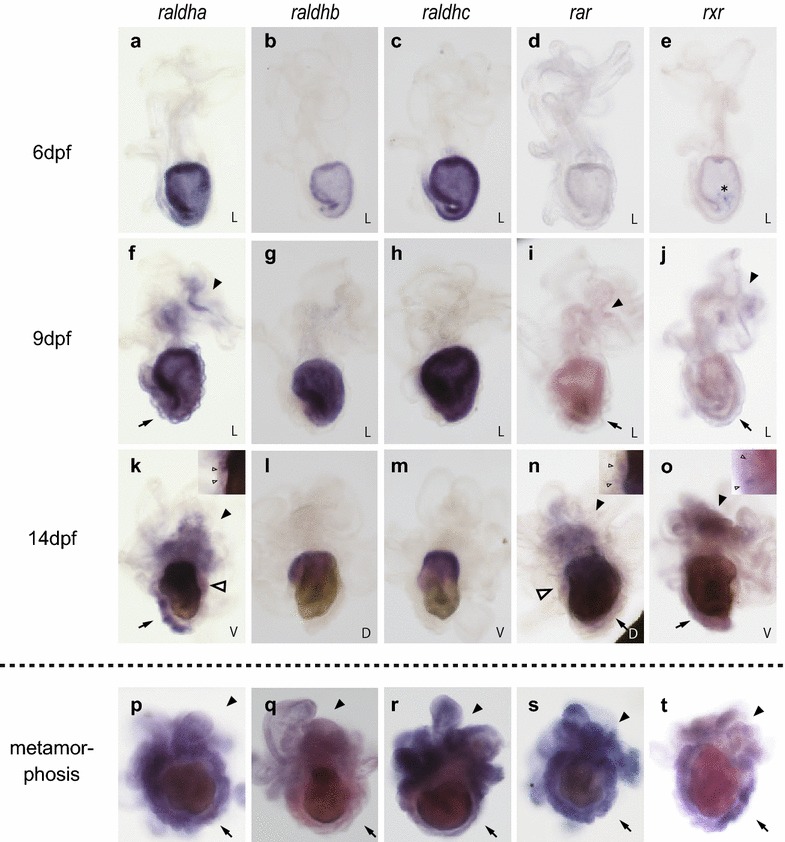
Spatial expression pattern of *raldha*, *raldhb*, *raldhc*, *rar*, and *rxr* in *P. pectinifera.*. **a**–**o** show results of whole mount in situ hybridization of *raldha*, *raldhb*, *raldhc*, *rar* and *rxr* in larval stages (**a**–**e** 6 dpf, **f**–**j** 9 dpf and **k**–**o** 14 dpf) and metamorphosing larvae just after settlement (**p**–**t**). Dot line means the boundary of before and after settlement. The inset figures of **k**, **n** and **o** show the expression in hydrolobes. L: left view, V: ventral view and D: dorsal view. Arrow heads indicate the expression in epidermis around brachiolar arms and white arrow heads point out the expression in hydrolobes. Arrows point expression in juvenile rudiment. Non-specific expressions are indicated by asterisk

We examined the spatial expression patterns of *raldha*, *raldhb*, *raldhc*, *rar*, and *rxr* by whole mount in situ hybridization in larvae of various stages (Fig. 5). *P. pectinifera* larvae became competent to metamorphose after 8 dpf, as shown previously. The expression of *raldha*, *raldhb*, and *raldhc* was detected in the foregut, midgut, and hindgut of larvae as young as 6 dpf, but no specific expression of *rar* or *rxr* was observed (Fig. 5a–e). In 9-dpf larvae that acquired competence, overlapping expression of *raldha*, *rar*, and *rxr* was detected in the juvenile rudiment and the epidermis around the brachiolar arms, whereas expression of *raldhb* and *raldhc* was restricted to the foregut, midgut and hindgut (Fig. 5f–j). Similar expression patterns were observed in 14-dpf larvae that did not receive environmental cues for metamorphosis, except that the expression of *raldha*, *rar* and *rxr* was also detected in the hydrolobes (Fig. 5k–o). In metamorphosing larvae after settlement, all genes that we examined were expressed in the whole epidermis of the larval body and in the juvenile rudiment (Fig. 5q–t). These expression patterns are consistent with our hypothesis that RA functions as an endogenous mediator of environmental cues, although an additional regulator seems to be required to activate RA signaling upon receiving cues, as discussed later in this paper.

## Discussion

### RA signaling acts as a regulator of starfish metamorphosis

**Fig. 6:**
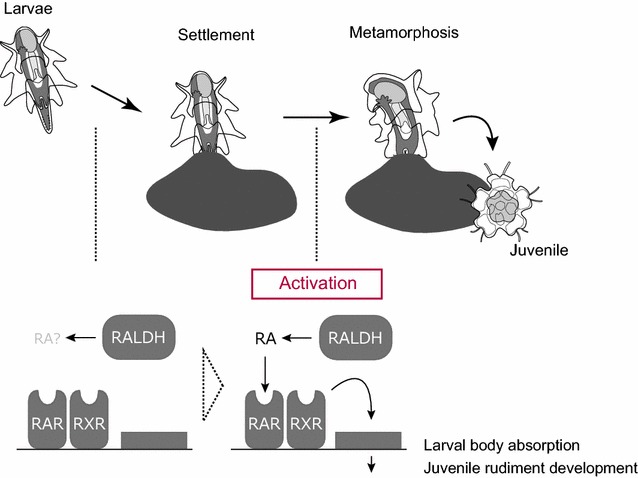
Schematic RA signaling function as regulator of metamorphosis. RA signaling activation is induced by receiving environmental cue with adhesive papillae of brachiolar arms and commences metamorphosis process

The life cycle transition from larva to juvenile in echinoderms occurs via two key steps. First, larvae acquire competence to metamorphose. Second, after receiving an environmental cue, metamorphosis proceeds with the absorption of the larval body and development of the pentaradial juvenile body. We present evidence that RA signaling functions as an endogenous mediator of environmental cues received upon attachment to a substrate in starfish (Fig. 6). Metamorphosis was suppressed by the inhibition of two distinct steps of RA signaling (Fig. 4): RA synthesis from retinal (by DEAB) and the reception of RA by RAR (by RO). We have not directly shown that RA is present in starfish larvae. Identification of three *raldh* genes, and the overlapping expression of one of them with *rar* and *rxr* in competent larvae (Fig. 5), however, strongly supports the idea that RA functions as an endogenous signaling molecule in starfish development.

Of note, the expression of *raldh*s, *rar*, and *rxr* was observed well before competent larvae received environmental cues (Fig. 5a–o). These expression patterns suggest the presence of another regulatory mechanism that controls the onset of RA signaling upon attachment. Murabe et al. indicated that environmental cues were received by adhesive papillae on the brachiolar arms [31]. Thus, the onset of RA signaling may be regulated by something in the adhesive papillae, likely through the nervous system. NO and HA are candidates for this regulator, as both have inhibitory effects on the onset of metamorphosis in sea urchins [11, 17, 24, 25]. Although no evidence of NO signaling or HA signaling has been reported for starfish, further studies on the crosstalk of these signals with RA signaling will facilitate a comprehensive understanding of the molecular regulatory mechanism of echinoderm metamorphosis.

### The role of the nuclear receptor/RXR heterodimer in metamorphosis regulation is evolutionarily conserved

RA signaling mediated by a RAR/RXR heterodimer receptor plays a critical role in the development of the unique body plans of chordates, such as the anterior–posterior patterning of the central nervous system [1, 2, 7]. Therefore, the ancestral role of RA signaling has attracted the interest of several zoologists [5, 6]. Our findings suggest that RA signaling plays a role in the regulation of metamorphosis in ancestral deuterostomes. During the evolution of ancestral chordates, RA signaling may have been co-opted for the patterning of the chordate body plan via the recruitment of *Hox* genes as regulators. Further investigation of RA signaling in non-chordate deuterostomes may shed new light on the evolution of the vertebrate body plan.

The transition of distinct body plans through metamorphosis is widely observed in metazoans [11, 41], and several recent studies have revealed that conserved molecular regulatory mechanisms underlie metamorphosis [10, 11, 42]. Molecular mechanisms for metamorphosis in amphibians and insects have been investigated in detail [12–16]. In both taxa, metamorphosis is regulated by hormones received by receptors (TH for amphibians and an ecdysone receptor for insects), which make a heterodimer with RXR. A recent study by Fuchs indicated that RXR is also involved in cnidarian metamorphosis [10]. Exogenous treatment with RA was shown to induce the metamorphic process of strobilation. Here, we present evidence that RA signaling is also involved in starfish metamorphosis, and provide additional evidence that components of RA signaling are conserved in this metamorphosis. However, amphibians and insects use different hormones for signaling, and RXR makes heterodimers with different counterparts accordingly. The suggested existence of a common ligand in cnidarians and starfish is thus intriguing and may reflect the ancestral molecular mechanism for metazoan metamorphosis. This hypothesis will be tested by further investigation of metamorphosis in various taxa, such as sea urchins, annelids and molluscs.

## Conclusions

We demonstrated that RA signaling performs as the regulator of metamorphosis process in echinoderm (starfish). Considering RA is also core element of metamorphosis regulation in basal group of metazoan (cnidarian), our results suggest that ancestral function of RA signaling is involved in metamorphosis.

## Additional files

**Additional file 1: Figure S1.** Phylogenic tree of Raldh (= Aldh1a, Aldehyde hydrogenase 1 alfa) and Aldh2 (Aldehyde hydrogenase 2) subfamily. The phylogenic tree was constructed by RAxML. *raldh*s of starfish made sister grouping with hemichordate *aldh1a*s. Numbers at node show bootstrap value. The selected amino acid substation model was LG + F + G. The process to construct was described in material and method and the set of sequences is provided in Additional file 10: Supplementary dataset 1. Abbreviations of species were referred following, Hs; *Homo sapiens* (Human), Mm; *Mus musculus* (Mouse), Xt; *Xenopus tropicalis* (Western clawed frog), Dr; *Danio rerio* (Zebrafish), Bf; *Branchiostoma floridae* (Amphioxus), Ci; *Ciona intestinalis* (Transparent sea squirt), Sk; *Saccoglossus kowalevskii* (Acorn worm), Sp; *Strongylocentrotus purpuratus* (Purple sea urchin), Pp; *Patiria pectinifera*.

**Additional file 2: Figure S2.** Phylogenic tree of RAR, RXR and THR (Thyroid hormone receptor). The phylogenic tree was constructed by RAxML. The selected amino acid substation model was LG + F + G. The set of sequences is provided in Additional file 11: Supplementary dataset 2. Abbreviations of species were referred following, Hs; *Homo sapiens* (Human), Mm; *Mus musculus* (Mouse), Xt; *Xenopus tropicalis* (Western clawed frog), Dr; *Danio rerio* (Zebrafish), Bf; *Branchiostoma floridae* (Amphioxus), Pm; *Polyandrocarpa misakiensis* (Tunicate), Bl; *Branchiostoma lanceolatum* (Amphioxus), Ci; *Ciona intestinalis* (Transparent sea squirt), Sk; *Saccoglossus kowalevskii* (Acorn worm), Sp; *Strongylocentrotus purpuratus* (Purple sea urchin), Pp; *Patiria pectinifera*, Dm; *Drosophila melanogaste*r (Fruit fly), Rc; *Reishia clavigera* (Sea snail), Ls; *Lymnaea stagnalis* (Great pond snail), Tc; *Tripedalia cystophora* (Jellyfish).

**Additional file 3: Table S1.** Accession numbers of the gene used for construction of phylogenic tree. Amino acid sequences to construct tree were obtained from Uniprot or Echinobase (http://www.echinobase.org/Echinobase/), Genbank.

**Additional file 4: Table S2.** Sequences of primer for amplification of *raldha*, *raldhb*, *raldhc*, *rar* and *rxr.* We used 40-bp reverse primers including a 20-bp T3 promoter sequence to synthesize Dig-labelled RNA probes for in situ hybridization. Bold characters mean consensus sequence for T3 promoter.

**Additional file 5: Table S3.** Number of metamorphosed/treated larvae of each batch in RO + RA treatment experiment.

**Additional file 6: Table S4.** Number of metamorphosed/settled larvae of each batch in RO 1 µM or DMSO treatment.

**Additional file 7: Table S5.** Number of settled larvae/treated larvae of each batch in RO 1 µM or DMSO treatment.

**Additional file 8: Table S6.** Number of settled/treated larvae of each batch in DEAB 100, 300 µM or DMSO treatment.

**Additional file 9: Table S7.** Number of metamorphosed/settled larvae of each batch in DEAB 100, 300 µM or DMSO treatment.

**Additional file 10: Supplementary dataset 1.** The dataset for phylogenetic analysis of Raldh and Aldh2.

**Additional file 11: Supplementary dataset 2.** The dataset for phylogenetic analysis of RAR, RXR and THR.
