# Supplementary material for: The role of retinoic acid signaling in starfish metamorphosis
Source: EvoDevo. 2018 Apr 21;9:10. doi: 10.1186/s13227-018-0098-x (PMC5910596; doi:10.1186/s13227-018-0098-x)
Supplement: Supplementary file 1 — Additional file 1: Figure S1. Phylogenic tree of Raldh (= Aldh1a, Aldehyde hydrogenase 1 alfa) and Aldh2 (Aldehyde hydrogenase 2) subfamily. The phylogenic tree was constructed by RAxML. raldhs of starfish made sister grouping with hemichordate aldh1as. Numbers at node show bootstrap value. The selected amino acid substation model was LG + F + G. The process to construct was described in material and method and the set of sequences is provided in Additional file 10: Supplementary dataset 1. Abbreviations of species were referred following, Hs; Homo sapiens (Human), Mm; Mus musculus (Mouse), Xt; Xenopus tropicalis (Western clawed frog), Dr; Danio rerio (Zebrafish), Bf; Branchiostoma floridae (Amphioxus), Ci; Ciona intestinalis (Transparent sea squirt), Sk; Saccoglossus kowalevskii (Acorn worm), Sp; Strongylocentrotus purpuratus (Purple sea urchin), Pp; Patiria pectinifera. [file 13227_2018_98_MOESM8_ESM.pdf]

number of settled / treated larvae

treatment	batch 1			batch 2			batch 3		
plate	1	2	3	1	2	3	1	2	3
DEAB,300μM	12/12	11/12	11/12	11/12	12/12	11/12	10/12	10/12	12/12
DEAB,100μM	12/12	9/12	12/12	12/12	12/12	12/12	11/12	9/12	10/12
DEAB,0μM	12/12	12/12	12/12	12/12	11/12	12/12	11/12	9/12	11/12
