# Supplementary material for: The role of retinoic acid signaling in starfish metamorphosis
Source: EvoDevo. 2018 Apr 21;9:10. doi: 10.1186/s13227-018-0098-x (PMC5910596; doi:10.1186/s13227-018-0098-x)
Supplement: Supplementary file 2 — Additional file 2: Figure S2. Phylogenic tree of RAR, RXR and THR (Thyroid hormone receptor). The phylogenic tree was constructed by RAxML. The selected amino acid substation model was LG + F + G. The set of sequences is provided in Additional file 11: Supplementary dataset 2. Abbreviations of species were referred following, Hs; Homo sapiens (Human), Mm; Mus musculus (Mouse), Xt; Xenopus tropicalis (Western clawed frog), Dr; Danio rerio (Zebrafish), Bf; Branchiostoma floridae (Amphioxus), Pm; Polyandrocarpa misakiensis (Tunicate), Bl; Branchiostoma lanceolatum (Amphioxus), Ci; Ciona intestinalis (Transparent sea squirt), Sk; Saccoglossus kowalevskii (Acorn worm), Sp; Strongylocentrotus purpuratus (Purple sea urchin), Pp; Patiria pectinifera, Dm; Drosophila melanogaster (Fruit fly), Rc; Reishia clavigera (Sea snail), Ls; Lymnaea stagnalis (Great pond snail), Tc; Tripedalia cystophora (Jellyfish). [file 13227_2018_98_MOESM9_ESM.pdf]

number of metamorphosed / settled larvae

treatment	batch 1			batch 2			batch 3		
plate	1	2	3	1	2	3	1	2	3
DEAB,300μM	0/12	0/11	0/11	3/11	5/12	4/11	0/10	0/10	0/12
DEAB,100μM	11/12	8/9	10/12	12/12	12/12	12/12	9/11	5/9	10/10
DEAB,0μM	10/12	12/12	9/12	12/12	11/11	12/12	11/11	9/9	11/11
