# Supplementary figures and images for: The role of retinoic acid signaling in starfish metamorphosis
Source: EvoDevo. 2018 Apr 21;9:10. doi: 10.1186/s13227-018-0098-x (PMC5910596; doi:10.1186/s13227-018-0098-x)

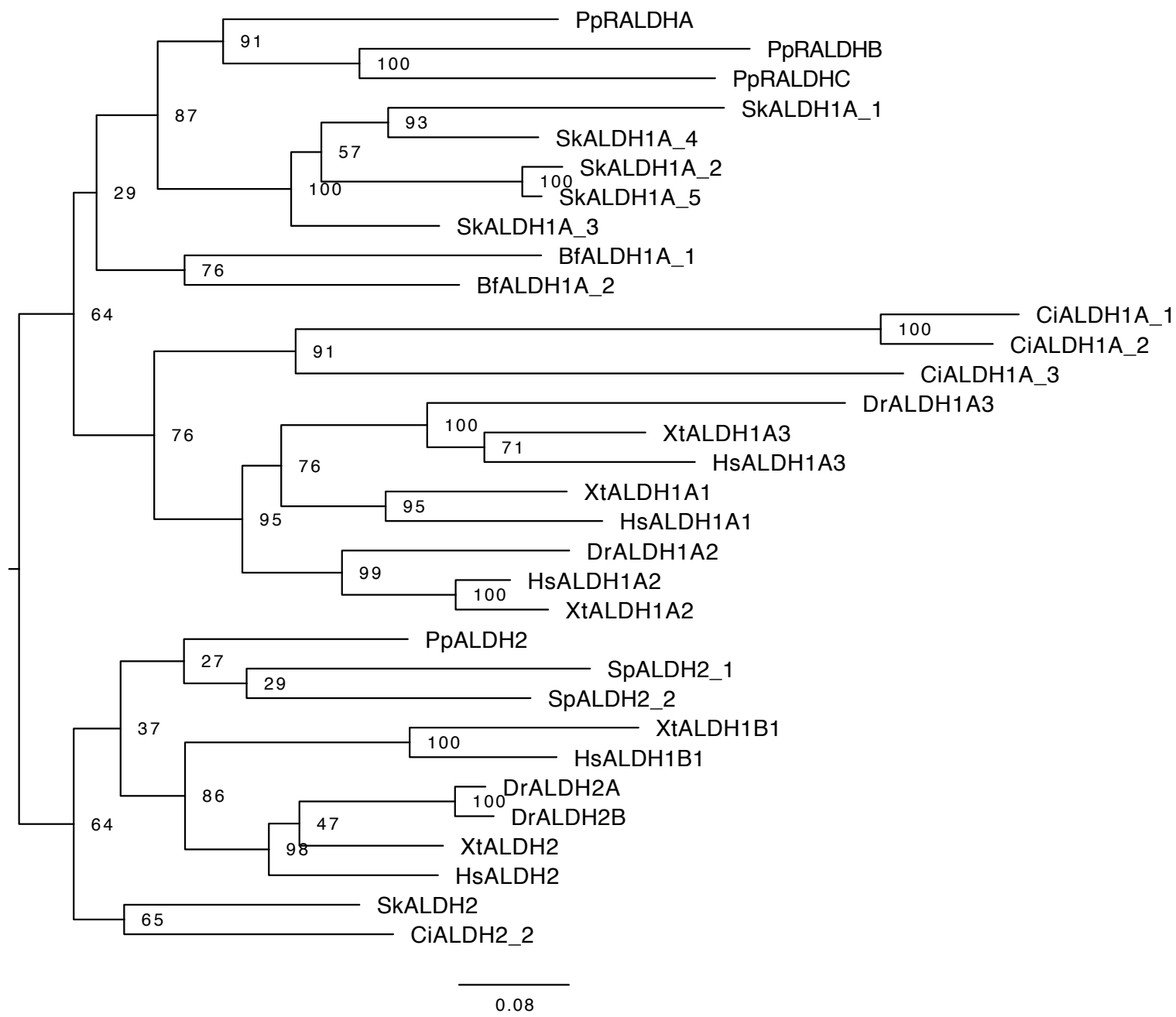

Supplement: Supplementary file 3 — Additional file 3: Table S1. Accession numbers of the gene used for construction of phylogenic tree. Amino acid sequences to construct tree were obtained from Uniprot or Echinobase (http://www.echinobase.org/Echinobase/), Genbank. [file 13227_2018_98_MOESM1_ESM.pdf]
