# Supplementary material for: The role of retinoic acid signaling in starfish metamorphosis
Source: EvoDevo. 2018 Apr 21;9:10. doi: 10.1186/s13227-018-0098-x (PMC5910596; doi:10.1186/s13227-018-0098-x)
Supplement: Supplementary file 5 — Additional file 5: Table S3. Number of metamorphosed/treated larvae of each batch in RO + RA treatment experiment. [file 13227_2018_98_MOESM3_ESM.pdf]

	gene/entry		gene/entry		
Hs	*aldh1a1*	P00352.2	*thra*	P10827	Hs; *Homo sapiens* (Human)
	aldh1a2	O94788	*thrb*	P10828	
	aldh1a3	P47895	*rara*	P10276	
	aldh1b1	P30837	*rarb*	P10826	
	aldh2	P05091	*rarg*	P13631	
			rxra	P19793	
			rxrb	P28702	
			rxrg	P48443	
Mm	*aldh1a1*	P24549	*thra*	P63058	Mm; *Mus musculus* (Mouse)
	aldh1a2	Q62148	*thrb*	P37242	
	aldh1a3	Q9JHW9	*rara*	P11416	
	aldh1b1	Q9CZS1	*rarb*	P22605	
	aldh2	P47738	*rarg*	P18911	
			rxra	P28700	
			rxrb	P28704	
			rxrg	P37238	
Xt	*aldh1a1*	Q4VBE1			Xt; *Xenopus tropicalis* (Western clawed frog)
	aldh1a2	Q9DEX5			
	aldh1a3	F7BV06			
	aldh1b1	F7DQF8			
	aldh2	Q6DJ49			
Dr	*aldh1a2*	Q90XS8	*thraa*	Q98867	Dr; *Danio rerio* (Zebrafish)
	aldh1a3	Q0H2G3	*thrab*	U3JAT9	
	aldh2a	Q8QGQ2	*thrb*	Q9PVE4	
	aldh2b	Q6TH48	*raraa*	Q90271	
			rarab	Q7ZTI3	
			rarga	Q91392	
			rargb	A2T928	
Bf	*aldh1a_1*	C3ZGK4	*rxr*	Q8MX78	Bf; *Branchiostoma floridae* (Florida lancelet) (Amphioxus)
	aldh1a_2	C3ZG63			
Bl			*rar*	O18608	Bl; *Branchiostoma lanceolatum* (Common lancelet)
Ci	*aldh1a_1*	A0A1W2WB51	*rar*	Q4H2W1	Ci; *Ciona intestinalis* (Transparent sea squirt)
	aldh1a_2	A0A1W5BCT1	*rxr*	Q4H2U9	
	aldh1a_3	A0A1W2WDC1			
	aldh2	A0A1W5B7N8			
Pm			*rxr*	K7ZLP3	Pm; *Polyandrocarpa misakiensis* (Tunicate)
Sk	*aldh1a_1*	XP_006823779.1	*rar*	XP_002742241.1	Sk; *Saccoglossus kowalevskii* (Acorn worm)
	aldh1a_2	XP_006822197.1	*rxr*	D2XNK4	
	aldh1a_3	XP_002736989.1			
	aldh1a_4	XP_002731204.1			
	aldh1a_5	XP_006824634.1			
	aldh2	XP_006816163.1			
Sp	*aldh2_1*	SPU_007284	*thr*	SPU_025239	Sp; *Strongylocentrotus purpuratus* (Purple sea urchin)
	aldh2_2	SPU_023801	*rar*	SPU_016523	
			rxr	SPU_028422	
Pp	*raldha*	LC379260	*rar*	LC379258	Pp; *Patiria pectinifera* (starfish)
	raldhb	LC379261	*rxr*	LC379259	
	raldhc	LC379262	*thr*	*	
	aldh2	*			
Dm			*usp*	P20153	Dm; *Drosophila melanogaster* (Fruit fly)
Rc			*rar*	T2HRZ4	Rc; *Reishia clavigera* (Sea snail)
			rxr	E9RHD8	
Ls			*rar*	D5LIR6	Ls; *Lymnaea stagnalis* (Great pond snail)
			rxr	Q5I7G2	
Tc			*rxr*	O96562	Tc; *Tripedalia cystophora* (Jellyfish)

* Sequences were not deposited to databank but available from supplementary dataset 1 or 2 of this work.
