# Supplementary material for: The role of retinoic acid signaling in starfish metamorphosis
Source: EvoDevo. 2018 Apr 21;9:10. doi: 10.1186/s13227-018-0098-x (PMC5910596; doi:10.1186/s13227-018-0098-x)
Supplement: Supplementary file 6 — Additional file 6: Table S4. Number of metamorphosed/settled larvae of each batch in RO 1 µM or DMSO treatment. [file 13227_2018_98_MOESM4_ESM.pdf]

	Forword Primer (5'→3')	Reverse Primer including T3 promoter region (5'→3')
raldha	CGAAATGGCTCCCCCACAAGTCAAGTACAC	**ATTAACCCTCACTAAAGGGA**AAATGCTCTCGGAAATTCAA
raldhb	CCGACGTGGATATTGCTGTGAATGCTGCAA	**ATTAACCCTCACTAAAGGGA**CTAGGCCCATCCAATCAATG
raldhc	AATGAGTTCGTCAATTCCGTGAGCGGAAAG	**ATTAACCCTCACTAAAGGGA**TTGGGAATGTTGCTTGAACC
rar	ATGAACATGAACATGAATCCTAATTACTCA	**ATTAACCCTCACTAAAGGGA**TTGGTTGTCGGCTCTGTCAA
rxr	AACTGCATCGTGGACAAACGTCAGAGGAAT	**ATTAACCCTCACTAAAGGGA**ACCACAGACTGTTCGGATAC
