# Supplementary material for: The role of retinoic acid signaling in starfish metamorphosis
Source: EvoDevo. 2018 Apr 21;9:10. doi: 10.1186/s13227-018-0098-x (PMC5910596; doi:10.1186/s13227-018-0098-x)
Supplement: Supplementary file 7 — Additional file 7: Table S5. Number of settled larvae/treated larvae of each batch in RO 1 µM or DMSO treatment. [file 13227_2018_98_MOESM5_ESM.pdf]

number of metamorphosed / treated larvae

treatment	batch 1		batch 2		batch 3	
RO, 3μM	+	−	+	−	+	−
RA, 0.1μM	6/20	18/20	0/20	17/20	0/20	17/20
RA, 1μM	15/20	20/20	2/20	20/20	1/20	18/20
