# Supplementary material for: The role of retinoic acid signaling in starfish metamorphosis
Source: EvoDevo. 2018 Apr 21;9:10. doi: 10.1186/s13227-018-0098-x (PMC5910596; doi:10.1186/s13227-018-0098-x)
Supplement: Supplementary file 8 — Additional file 8: Table S6. Number of settled/treated larvae of each batch in DEAB 100, 300 µM or DMSO treatment. [file 13227_2018_98_MOESM6_ESM.pdf]

number of metamorphosed / settled larvae						
treatment	batch 1		batch 2		batch 3	
plate	1	2	1	2	1	2
RO, 1μM	0/12	0/12	1/9	1/11	1/12	4/12
RO, 0μM	12/12	12/12	12/12	11/11	11/12	12/12
