# Supplementary material for: The role of retinoic acid signaling in starfish metamorphosis
Source: EvoDevo. 2018 Apr 21;9:10. doi: 10.1186/s13227-018-0098-x (PMC5910596; doi:10.1186/s13227-018-0098-x)
Supplement: Supplementary file 4 — Additional file 4: Table S2. Sequences of primer for amplification of raldha, raldhb, raldhc, rar and rxr. We used 40-bp reverse primers including a 20-bp T3 promoter sequence to synthesize Dig-labelled RNA probes for in situ hybridization. Bold characters mean consensus sequence for T3 promoter. [file 13227_2018_98_MOESM2_ESM.pdf]

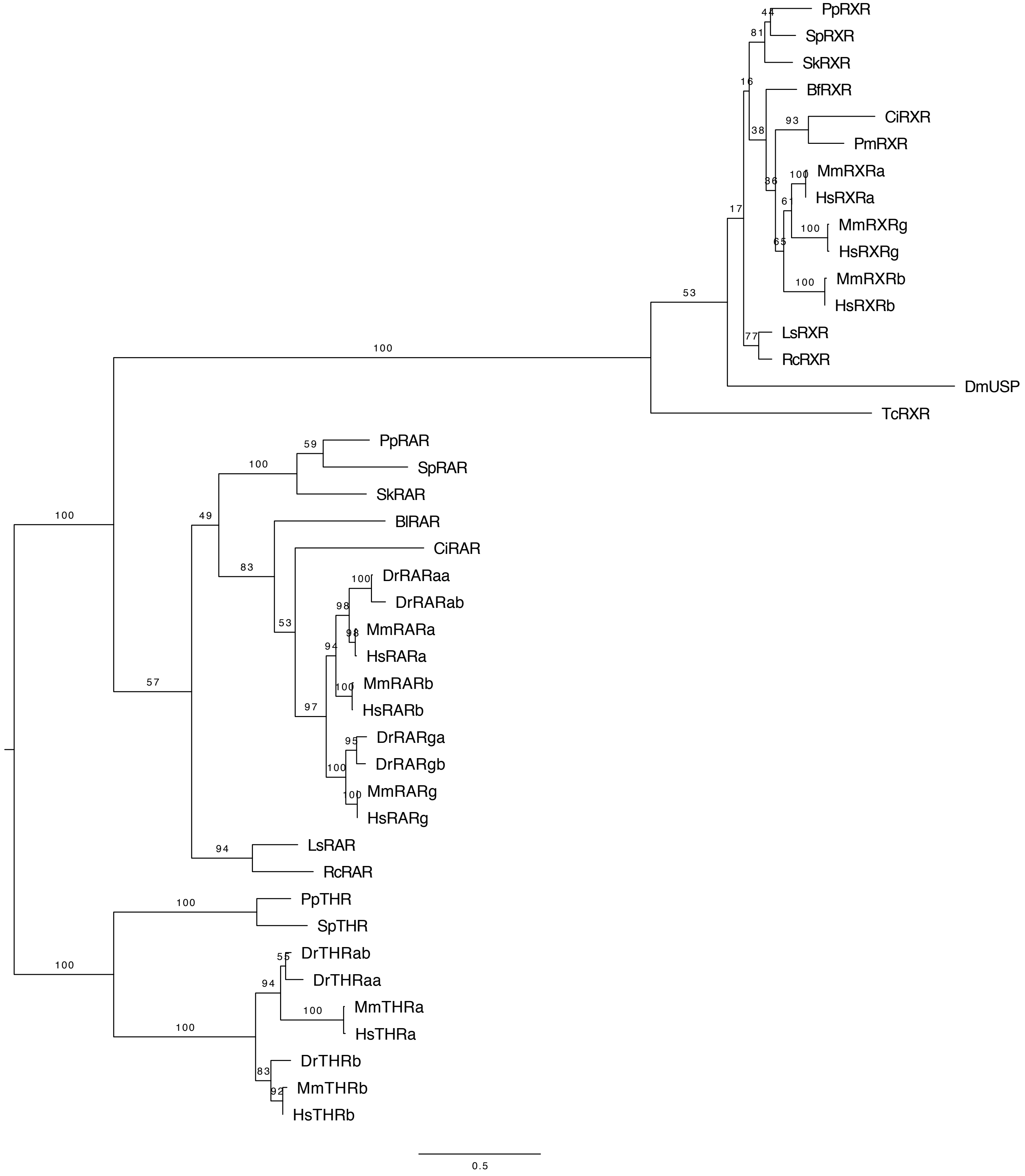

PpRXR
SpRXR
SkRXR
BfRXR
CiRXR
PmRXR
MmRXRa
HsRXRa
MmRXRg
HsRXRg
MmRXRb
HsRXRb
LsRXR
RcRXR
DmUSP
TcRXR
PpRAR
SpRAR
SkRAR
BlRAR
CiRAR
DrRARaa
DrRARab
MmRARa
HsRARa
MmRARb
HsRARb
DrRARga
DrRARgb
MmRARg
HsRARg
LsRAR
RcRAR
PpTHR
SpTHR
DrTHRab
DrTHRaa
MmTHRa
HsTHRa
DrTHRb
MmTHRb
HsTHRb
0.5
